# Novelty detection in rover-based planetary surface images using autoencoders

**DOI:** 10.3389/frobt.2022.974397

**Published:** 2022-10-14

**Authors:** Braden Stefanuk, Krzysztof Skonieczny

**Affiliations:** Aerospace Robotics Laboratory, Electrical and Computer Engineering, Concordia University, Montreal, QC, Canada

**Keywords:** autoencoder, novelty detection, planetary science, planetary rovers, precision and recall, dimensionality reduction

## Abstract

In the domain of planetary science, novelty detection is gaining attention because of the operational opportunities it offers, including annotated data products and downlink prioritization. Using a variational autoencoder (VAE), this work improves upon state-of-the-art novelty detection performance in the context of Martian exploration by >7% (measured by the area under the receiver operating characteristic curve (ROC AUC)). Autoencoders, especially VAEs, perform well across all classes of novelties defined for Martian exploration. VAEs are shown to have high recall in the Martian context, making them particularly useful for on-ground processing. Convolutional autoencoders (CAEs), on the other hand, demonstrate high precision making them good candidates for onboard downlink prioritization. In our implementation adversarial autoencoders (AAEs) are also shown to perform on par with state-of-the-art. Dimensionality reduction is a key feature of autoencoders for novelty detection. In this study the impact of dimensionality reduction on detection quality is explored, showing that both VAEs and AAEs achieve comparable ROC AUCs to CAEs despite observably poorer (blurred) image reconstructions; this is observed both in Martian data and in lunar analogue data.

## 1 Introduction

In the past 20 years, exploration missions to the Moon and Mars^1^ have borne abundant, high-resolution images (among other data products) to the research community, paving the way for future exploration to be conducted more autonomously and more robustly. Several factors are increasingly driving the need for autonomy in planetary science operations. In Mars rover operations, visual surface investigation and decision-making currently takes place in day-long tactical cycles with a desire for faster turnaround Gaines et al. (2016). Upcoming commercial lunar rover missions will have short lifetimes and severely constrained bandwidth shared across several payloads. This will result in a need for rapid tactical decision-making processes with limited or even omitted data Raimalwala et al. (2020). In the domain of planetary exploration, bandwidth is a scarce resource and high cadence telemetry is not always (perhaps even rarely) practical. Autonomous onboard novelty detection offers a way to prioritize data downlink and, in the future, achieve autonomous science targeting altogether.

Novelty detection is part of a well-established set of techniques used to detect samples or features from within a set of data that are either unique or statistically uncommon. In nomenclature, anomaly detection is an umbrella term that covers both outlier detection and novelty detection. Although the definitions of these methods differ slightly, it is often appropriate to use them interchangeably (Xu et al., 2019). Autoencoders have been applied to the problem of novelty detection since the early 2000s (Thompson et al., 2011). However, interest in the approach picked up in the mid 2010s when several teams released research demonstrating the applicability of autoencoders to image-based novelty detection (Pimentel et al., 2014; Chen et al., 2017).

Extensions to the standard fully convolutional autoencoder (CAE) have been proposed that use probabilistic encoders and decoders—that is, they output parameters of the encoding distribution instead of the encoded pixel values themselves. Two primary extensions have been proposed (An and Cho, 2015; Leveau et al., 2017; Beggel et al., 2019):

1) Variational autoencoders (VAEs) leverage the Evidence Lower Bound and KL-divergence to map the latent space representation to a prior distribution, typically a unit Gaussian (Doersch, 2016). The latent representation is found by sampling from the encoded distribution, while the final data product is obtained by decoding the latent representation back to the original dimensionality.
2) Adversarial autoencoders (AAEs) leverage an adversarial procedure to obtain reconstructions. They calibrate the aggregated posterior of the latent distribution by matching it to an imposed prior distribution (Makhzani et al., 2015).

Novelty detection as applied to the domain of planetary exploration images was spearheaded by Kerner et al. (2018) and Kerner et al. (2020). These works established a dataset of the Martian terrain for the purpose of developing and testing novelty detection algorithms. They implemented and analyzed a swath of techniques, including Principal Component Analysis (PCA), Reed-Xiaoli (RX) detectors, Generative Adversarial Networks (GANs), and CAEs. Various loss functions and novelty scores were used to compare the advantages and disadvantages of each detector. It was determined that, while autoencoders only performed on par or marginally better than other methods, they were easier to visualize and thus could be used to add more meaningful context to detections than alternative approaches. Since the training objective of an autoencoder is to reproduce the input image from a lower dimensional (latent) representation, once an autoencoder has been trained, error maps can be created to visualize how the reconstructed outputs and the original inputs differ. These error maps supply spatial and spectral information about the location and magnitude of novel features.

The main contributions of this paper include autoencoder implementations that outperform state-of-the-art methods by >7% on a dataset from NASA’s Curiosity rover’s Mastcam. We also introduce the first (known) implementation of both a VAE and an AAE to the field of rover-based planetary novelty detection and gauge their performance against CAEs. We identify specific operational applications where the different types of autoencoders are most useful by investigating their predictive capabilities in terms of recall and precision, probability of detection, and statistical metrics for model selection, when possible expanding the analysis to the viability of such models in an operational setting. We introduce a new metric, precision at capacity, for novelty detection in bandwidth-constrained applications. Details of algorithmic methodology and a code repository are also contributed.

This study represents strides that have been made to date towards an end-to-end detector that can be integrated into future rover missions on the systems level. It builds significantly upon prior work published by the authors (Stefanuk et al., 2020), and comprises an important part of the first author’s MASc thesis (Stefanuk, 2021), which also includes additional related work that may be of interest to the reader.

## 2 Materials and methods

This research is in the domain of machine learning for image processing. The relevant methodological elements for such problems include the datasets, computing hardware and software, and algorithmic implementation details.

### 2.1 Datasets

The results of this research are based principally upon data from the Curiosity rover operating on the Martian surface. However, an analogue lunar dataset that was developed as a collaborative effort between the authors and Mission Control Space Services is used to further investigate key observations.

#### 2.1.1 Curiosity mastcam

**FIGURE 1:**
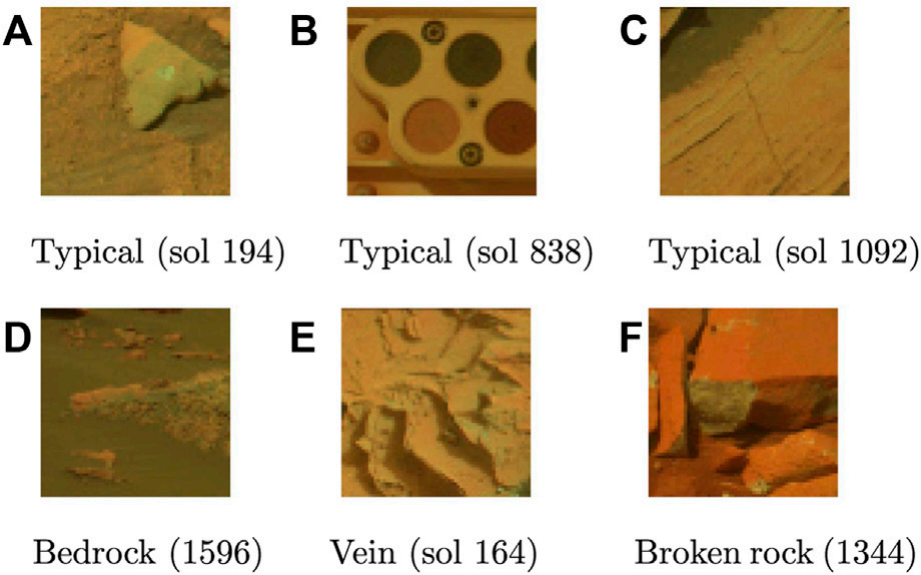
Images from the Curiosity Mastcam dataset; **(A–C)** shows typical images, **(D–F)** shows novel images. All images in this dataset have a height and width of 64 pixels with six spectral channels. For viewing purposes, only the first three channel are shown.

This dataset—targeted for the problem of novelty detection on the Martian surface—was introduced and then later updated by Kerner et al. (2018) and Kerner et al. (2020); the updated form is the one used herein. Images for this dataset are sourced directly from data collected by the Mars Science Laboratory (MSL) Curiosity rover’s mast camera (Mastcam). The Mastcam is equipped with two focusable CCD cameras and is capable of capturing stereoscopic, multi-spectral images. Both cameras sport a filter wheel with 8-channels, six of which are narrow-band filters ranging from 400 to 1,100 nm (Bell et al., 2017). To create the dataset, all images acquired with the six narrow-band filters from sols (Martian days) 1–1,666 were considered. Since the first image products available on-ground during MSL operations are the uncalibrated thumbnails of full-resolution images, expert analysts inspected these thumbnails for the presence of novelties (Kerner et al., 2020). If found, a novelty was annotated with a 64 × 64 pixel bounding box. Using a sliding window approach, each image in the set of available images (477 in total) was cropped into 64 × 64 pixel sub-images (Figure 1). After this process was complete, 9,302 and 1,386 typical images were allocated to the training and validation sets respectively. The test set consists of 426 typical images and 430 novel images.

Eight novelty classes were used for this dataset: DRT spot, dump pile, broken rock, drill hole, meteorite, vein, float rock, and bedrock. While the first four classes are created by the rover itself, the remaining four classes are due to natural processes on Mars: *meteorites* are sporadically found by Martian rovers and serve as either obstacles or scientific targets; *bedrock* enables elemental analysis of the underlying material; *veins* are hard-packed granules that fill fractures through a variety of processes, and *float rocks* are detached rock fragments that have been transported due to geologic activity.

While the Curiosity Mastcam dataset is valuable in terms of its ability to test the behaviour of novelty detection routines in for planetary exploration, it simplifies many intricacies that must be resolved to use novelty detection for autonomous science in an operational setting. This dataset is used in this research as a benchmark in the context of planetary exploration. One advantage that this dataset offers is that each image has six multispectral channels, allowing insight into the advantages provided by greater spectral resolution for novelty detection.

Since this dataset was prepared for the research community, only minor preprocessing need to be conducted. First, each image was converted from unsigned integer data type with intensities between 0 and 255 into 32-bit floating-point values, then they were standardized to mean zero and unit standard deviation before being used for training, validation, and testing.

#### 2.1.2 Lunar analogue

The Lunar Analogue dataset developed and presented in (Stefanuk et al., 2021) was also used in this research. In contrast to the Curiosity Mastcam dataset, which is already curated into small selected patches, this Lunar Analogue dataset starts with high-resolution images of whole landscapes. Due to the nature of the Lunar Analogue dataset it was important to implement a preprocessing pipeline that resulted in images that were as robust as possible to illumination conditions, imaging perspectives, and different levels of zoom. First, the full-resolution images (1,242 × 2,208) were resampled using pixel-area relocation to a resolution of 248 × 441, one-fifth the original size; the aspect ratio was retained in this process. Histogram equalization was then conducted by first converting the RGB image into YCrCb format, equalizing on the intensity channel, and converting back to RGB. Minor median blurring with a 3 × 3 kernel was conducted and images were standardized to zero mean with unit standard deviation.

From a lunar geology perspective, any terrain feature that can be used to trace the history of the region is of interest. For example, features with steep inclines, such as lava channels or craters walls, can provide access to the lunar bedrock where spectroscopic methods can be used to characterize the composition of the substratum. In certain areas of the Moon that were prone to volcanic activity, dark-toned cobbles can be found that inform scientists about the region’s volcanism. Similarly, fragments of mantle rock that are encased within larger rocks formed through volcanic activity. Light-toned ejecta that extend from craters (crater rays) can be helpful when inferring the approximate age of the crater. There also exist cases of pyroclastic deposits that appear following fountain-style volcanic eruptions, they have been documented to exhibit green and orange hues NASA (2017). With consultation and validation from lunar geologists (Stefanuk et al., 2021), Table 1 outlines the novelty labels used in the dataset.

**TABLE 1:** List of novelties used in Lunar Analogue dataset.

Novelty	Description
Rille	Channel-like depression in the terrain, formed by old lava flows.
Volcanic rock	Either (i) rocks with dark outer crust (fusion crust), or (ii) smaller fragments of mantle encased in larger rocks, formed during volcanic activity (mantle xenolith).
Exposed bedrock	Visible underlying substratum surrounded by regolith.
Green soil	Pyroclastic deposit exhibiting green hues, produced by fountain-style eruptions.
Orange soil	Pyroclastic deposit exhibiting orange hues, produced by fountain-style eruptions.

All novelties are labeled with bounding box labels and all typical images are unlabelled (the lack of label explicitly indicating that they are entirely typical). In this dataset, 4,809 images are used for training and validation, 854 are used for testing, of which 426 are typical and 428 contain at least one novelty.

Within the scope of this present study, the intent is to identify whether novelties are present in small images cropped from landscapes. Such a dataset was not explicitly prepared from the Lunar Analogue data. One could imagine applying the windowed cropping method used in the Curiosity Mastcam dataset (Kerner et al., 2020), or applying warping to the already-labeled novelty bounding boxes within the Lunar Analogue data (alongside randomly selected boxes within unlabeled typical regions of the landscape images). On the other hand, the authors had already generated useful data in the course of doing separate research on unsupervised intelligent novel region extraction from the Lunar Analogue landscape images. As the Lunar Analogue data is not the principal focus in this work, but rather is used for secondary support of some observations, such existing data is sufficient for our purposes.

**FIGURE 2:**
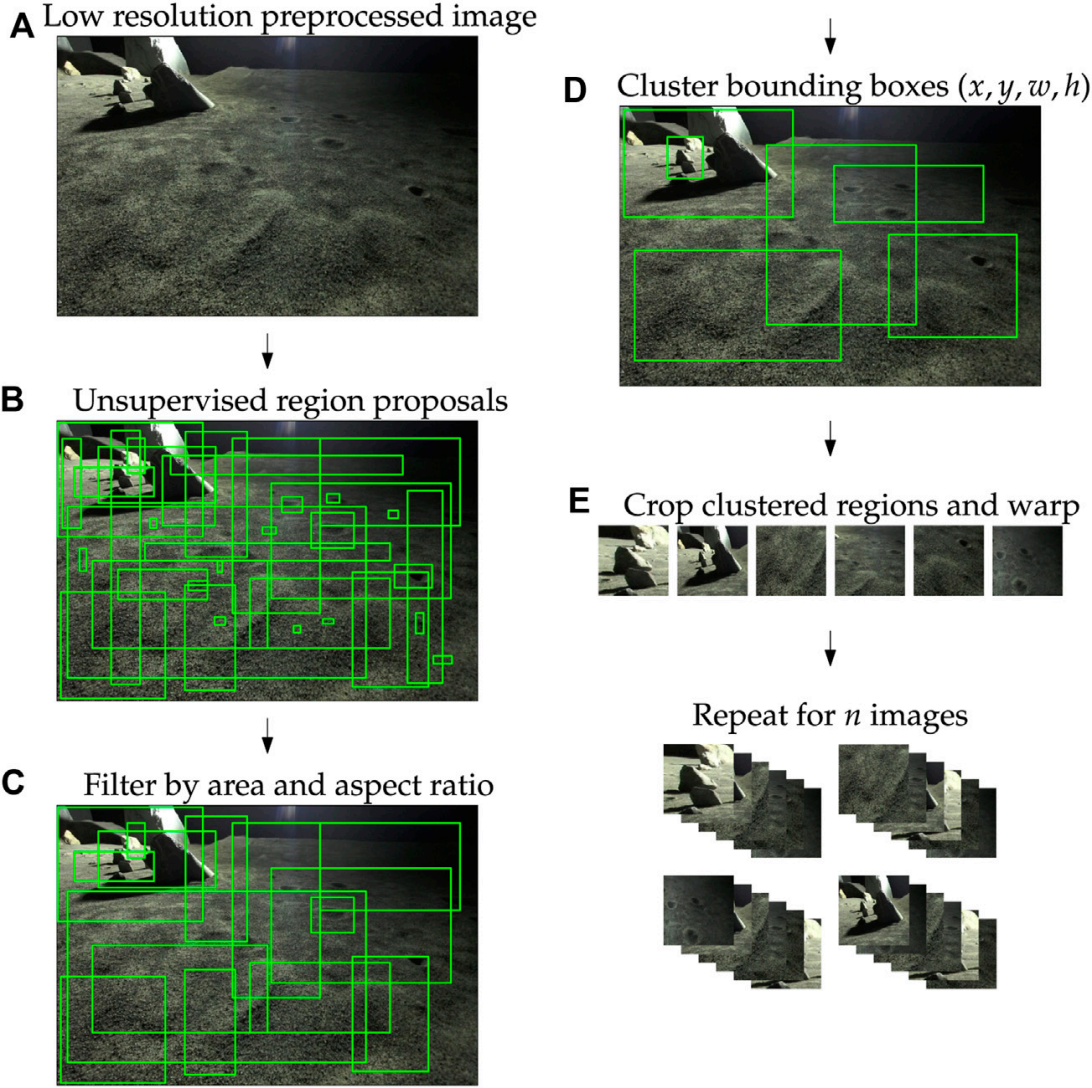
**(A–E)** Composition of the region proposal system used for novel region extraction.

**FIGURE 3:**
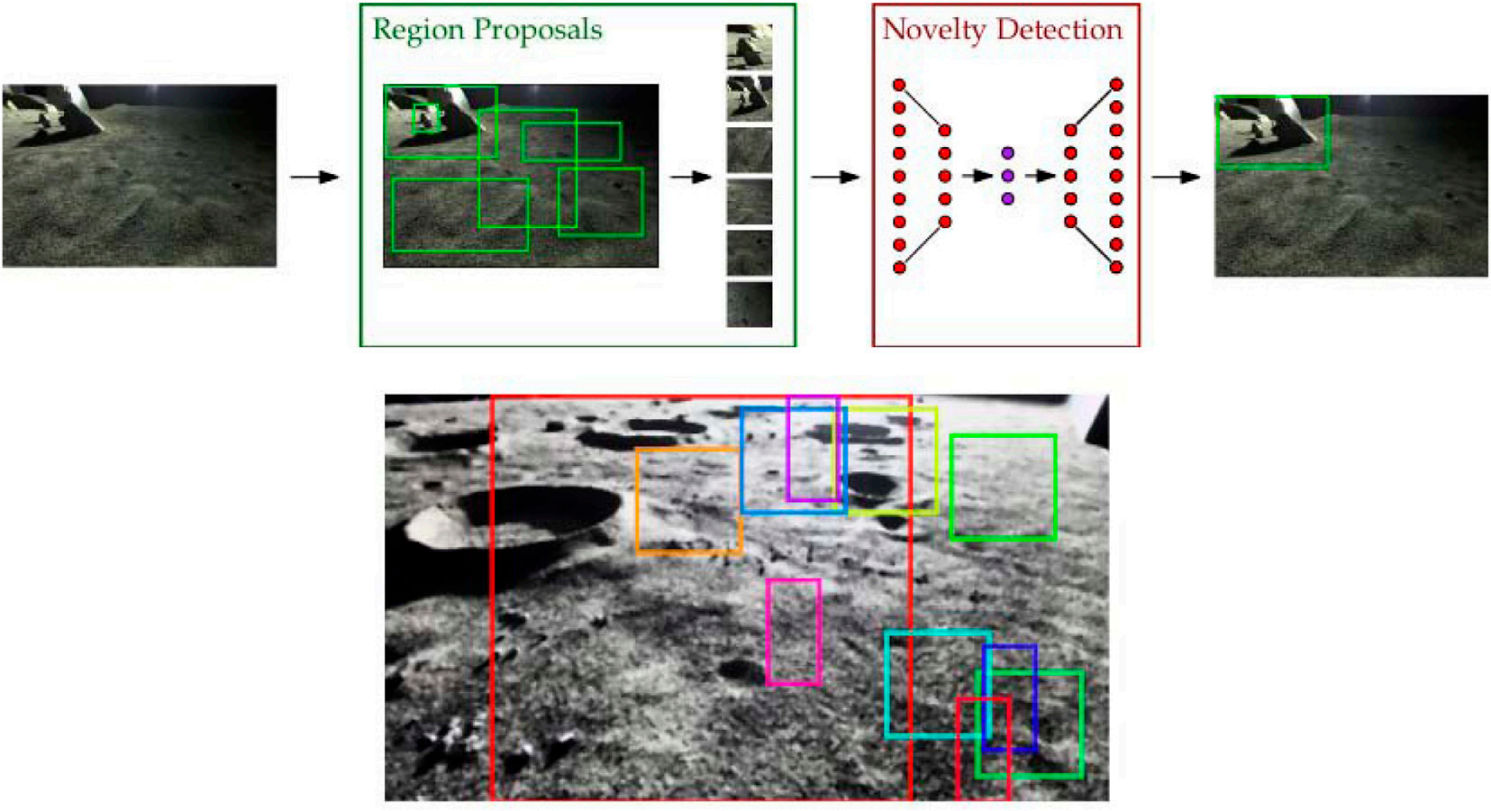
(*top*) Schematic of the novel region extraction (NRE) pipeline. NREs cascade a class-agnostic region proposal system with full-image novelty detection and can be trained like traditional reconstruction-based novelty detectors. (*bottom*) Sample of raw, unfiltered region proposals by the Binarized normed gradients (BING) method.

For completeness, the process by which the Lunar Analogue cropped regions were produced is described here, and shown in Figure 2. Region proposals within a landscape image of the Lunar Analogue data were based on saliency detection using the binarized normed gradients (BING) method, pre-trained on PASCAL VOC 2007 to be receptive to generic image features such as edges and textures (Cheng et al., 2019). This algorithm was designed for high frame-rate saliency detection in videos, so it is well-suited for the domain of planetary exploration where computational cost is a primary concern. Examples of region proposals resulting from BING are shown in Figure 3.

Regions with small sizes or large aspect ratios were removed, as shown in Figure 2C. Since the regions extracted from the system are warped before being used for training, it was important to imbue some sense of consistency into the data. Regions with large aspect ratios, upon warping, would fail to resemble actual objects within the scene. Similar reasoning led to the exclusion of small bounding boxes. Small areas below the 90th percentile were filtered as were large aspect ratios (most-elongated in either height:width or its inverse) above the 70th percentile; these percentiles were held as controls. After the filtering step, *k*-means clustering was used to maximize image coverage, minimize bounding box overlap, and limit the proposals to a static number of regions. To achieve this, clustering was done on 4-dimensional parameter space consisting of the *x*, *y* positions (top left) of each proposed region’s bounding box as well as the box’s width *w* and height *h*. This effectively clustered regions based on both their size and position within the image. Once the clusters were fit, the cluster centroids (*x*_*i*_, *y*_*i*_, *w*_*i*_, *h*_*i*_) were used as the final region proposals. For the BING-RP system, 16 clustered regions were used as we found that this to be a good control that managed both image coverage and computer resources well. The final step was to crop each region, warp them to a size of (3, 64, 64), and pass them on to the different novelty detection algorithms.

Implementing *k*-means clustering into the overall region proposal system was most important when preparing the data for training. Since the regions are cropped and warped directly from the output of the region proposal system, it was in our interest to eliminate redundant training samples and reduce overlap between extracted regions.

### 2.2 Hardware and software

All software used in this paper can be found in the companion repository at github.com/brahste/novelty-detection. Our autoencoder models were built with PyTorch while PCA was implemented with Scikit-Learn. All models were trained on a single GTX 1080 Ti graphical processing unit (GPU).

### 2.3 Algorithmic implementations

The novelty detection algorithms presented here are all reconstruction-based, meaning that the original image is compressed into a latent (reduced-dimension) representation and reconstructed, and then the reconstructed image is compared to the original input. Novelties are defined by a threshold in a metric comparing reconstructed and original images, in this case the mean squared error (MSE). The algorithmic differences depend on the details of the dimensionality reduction and reconstruction, i.e. the architectures of the autoencoders (or other analogous algorithm) used.

#### 2.3.1 Principal component analysis

Principal Component Analysis (PCA) is treated first because it a well-established linear transformation that can be used for dimensionality reduction and reconstruction. It differs in nature from the nonlinear autoencoder-style novelty detection algorithms that form the predominant point of analysis in this research. PCA serves primarily as a benchmark to monitor the performance of other architectures discussed throughout this research. Our PCA implementation applied to Curiosity Mastcam datasets employs a conventional, whole-dataset approach that encodes all of the dimensions of the input dataset. For a training set X and a learned representation Y , both of size *m*, and each sample having dimensions *d* = *w* × *h* × *c*, PCA is applied pixel-wise on flattened images such that the learned transformation maps $T:X_{m\times d}\to Y_{m\times d}$ .

Subsequently, we truncate the learned set at the *k*th principal component. Given that the first principal component (PC) explains the most variance in the data, followed by the second, and so on, we dynamically select the value of *k* by using only the first components needed to explain 60% of the variance. Using the learned representation from the retained PCs, we evaluated the reconstructions on the test set and scored each sample as the mean squared error between the input and reconstruction.

It is worth mentioning that the choice to retain 60% of the components was made heuristically based on benchmarking evaluations. These evaluations and other detailed training information for all PCA models can be found in the logs at the companion repository under the github.com/brahste/novelty-detection.

#### 2.3.2 Convolutional autoencoder architectures

**FIGURE 4:**
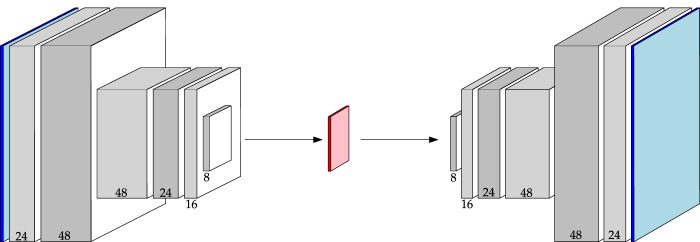
Architecture of the Baseline CAE. Channel numbers are shown at the bottom of each block. Reference Table 2 for a detailed description.

It is valuable to understand the behaviour of a purely convolutional autoencoder (CAE); CAEs are useful in their own right, and convolutional operations are fundamental to the structure of more advanced autoencoders as well. With this goal, two CAE architectures are explored in this study. The first architecture, the Baseline CAE, expands and deepens the network from (Kerner et al., 2020), as well as adopts alternative normalization routines. The Baseline CAE marks the primary convolutional architecture against which more advanced networks are measured. Figure 4 displays the morphology of an image from input to output through the Baseline CAE along with the channel depths resulting from each convolutional layer. The latent representation is a 3-channel feature map that acts as the bottleneck between the encoder and decoder. The Baseline CAE can be used for inputs of various size and channel depth, but in this study we focus on 64 × 64 images (principally 6-channel Curiosity Mastcam data, but also 3-channel Lunar Analogue Region images). A further summary of key architecture parameters is provided in Table 2.

**TABLE 2:** Summary of autoencoder architectures. C denotes the number of output channels.

Architecture	Layer	Kernel size	Stride	Dropout?	BN?	Activation?
Baseline CAE/VAE/AAE	Enc-1	24, 5, 5	1	✓	✓	✓
	Enc-2	48, 5, 5	1	✓	✓	✓
	Enc-3	48, 5, 5	2	✓	✓	✓
	Enc-4	24, 5, 5	1	✓	✓	✓
	Enc-5	16, 5, 5	1	✓	✓	✓
	Enc-6	8, 5, 5	2	✓	✓	✓
	Enc-7	3, 5, 5	1	✗	✗	✗
	Dec-1	8, 5, 5	1	✓	✓	✓
	Dec-2	16, 5, 5	2	✓	✓	✓
	Dec-3	24, 5, 5	1	✓	✓	✓
	Dec-4	48, 5, 5	1	✓	✓	✓
	Dec-5	48, 5, 5	2	✓	✓	✓
	Dec-6	24, 5, 5	1	✓	✓	✓
	Dec-7	C, 5, 5	1	✗	✗	✓
Compression CAE (MidCapacity)	Enc-1	24, 3, 3	1	✓	✓	✓
	Enc-2	24, 5, 5	1	✓	✓	✓
	Enc-3	48, 3, 3	2	✓	✓	✓
	Enc-4	48, 5, 5	1	✓	✓	✓
	Enc-5	24, 3, 3	2	✓	✓	✓
	Enc-6	24, 5, 5	1	✓	✓	✓
	Enc-7	8, 3, 3	2	✓	✓	✓
	Enc-8	1, 3, 3	1	✗	✗	✗
	Dec-1	8, 3, 3	1	✓	✓	✓
	Dec-2	24, 3, 3	2	✓	✓	✓
	Dec-3	24, 5, 5	1	✓	✓	✓
	Dec-4	48, 3, 3	2	✓	✓	✓
	Dec-5	48, 5, 5	1	✓	✓	✓
	Dec-6	24, 3, 3	2	✓	✓	✓
	Dec-7	24, 5, 5	1	✓	✓	✓
	Dec-8	C, 3, 3	1	✗	✗	✓
Simple VAE	Enc-1	16, 3, 3	2	✗	✓	✓
	Enc-2	32, 3, 3	2	✗	✓	✓
	Enc-3	64, 3, 3	2	✗	✓	✓
	Enc-***μ*** (Dense)	64 × $C_{e}^{2}$	NA	✗	✗	✗
	Enc-**Σ** (Dense)	64 × $C_{e}^{2}$	NA	✗	✗	✗
	Dec-**z** (Dense)	64 × $C_{e}^{2}$	NA	✗	✗	✗
	Dec-1	32, 3, 3	2	✗	✓	✓
	Dec-2	16, 3, 3	2	✗	✓	✓
	Dec-3	16, 3, 3	2	✗	✓	✓
	Dec-4	C_**x**_, 3, 3	2	✗	✓	✓

During the prototyping phase of this research, it was found that the extent of compression between the input and latent space affected the ability of CAE networks to infer novelty. Networks that lose little information during the encoding process have the potential to reconstruct both training and testing data in too much detail, resulting in poor novelty detection. In response to this insight, another network was built to further investigate the implications of dimensionality reduction and overall network capacity. This network, named CompressionCAE-MidCapacity, shares the same general architecture as Baseline CAE, but differs in the size of the convolutional kernels, and thus the network capacity. Most notably, it contains an extra interpolation step compared to the Baseline CAE, thus boosting the extent of compression between the input and latent space. A further summary of key architecture parameters is provided in Table 2.

Both CAE architectures share a number of attributes that were either held static between experiments and training sessions, acting as controlled variables, or manipulated as a (hyper)parameter of the experiment. A Leaky Rectified Linear Unit (Leaky ReLU) with a leak rate of 0.1 was used to activate non-linearities in the main encoding and decoding blocks for all CAE architectures. For those layers that used batch normalization and drop out, a default momentum and drop rate of 0.1 were used respectively. Mild variations to these default values were used in some experiments. No activations, batch normalization, or dropout were used at the encoder output. The decoder output was transformed by a hyperbolic tangent operation such that the resulting data would be constrained within the range [-1,1]. For training, an automatic learning rate determination algorithm proposed by Smith (2017) was used. Some manually selected learning rates were also used to assess performance changes against the learning rate that was automatically computed. Mild weight decay was employed in all CAE architectures using a tuning parameter of 0.01. Batch sizes varied between experiments, ranging between 8 and 1,024 images per batch. In all experiments 20% of the training data was allocated for validation.

Detailed training information for all CAE models can be found in the logs at the companion repository at github.com/brahste/novelty-detection.

#### 2.3.3 Variational autoencoder architectures

**FIGURE 5:**
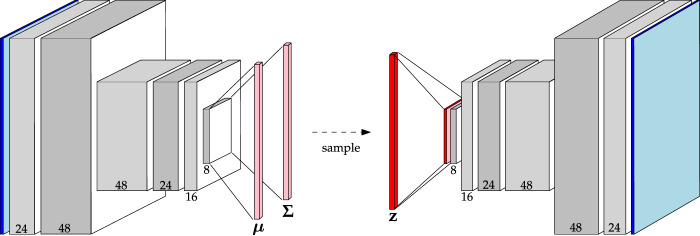
Architecture of the Baseline VAE. Channel numbers are shown at the bottom of each block. Reference Table 2 for a detailed description.

Variational autoencoders extend the interpretation of CAEs to probabilities by adding a KL-divergence penalty on the latent distribution. To explore the utility of VAEs for planetary novelty detection, two architectures were built and trained. The first uses the same set of convolutional layers outlined in Table 2. This model is aptly called Baseline VAE. By sharing the same convolutional structure as other Baseline models, a control is added to the experiments such that a more meaningful comparison can be conducted between the two algorithms. The Baseline VAE is shown in Figure 5 where one can see that the encoder computes the parameters ***μ***, **Σ** of the latent distribution, which is sampled to obtain the latent representation itself.

Also, to expand upon the VAEs that we implemented in the early stages of this research (Stefanuk et al., 2020), we investigate the Simple VAE architecture as also outlined in Table 2. While this architecture also uses convolutional operations in the encoder and decoder, the feature maps are flattened to obtain the parameter vectors ***μ***, **Σ** and subsequent latent representation **z**. One key difference between the Baseline VAE and Simple VAE is that the Simple variant has a lower capacity. To accommodate this difference the Simple VAE was not regularized with dropout layers, though weight decay regularization was retained.

Similar hyperparameters were used for all VAEs, barring the use of dropout layers as described above. For those layers that do use dropout, a dropout of 0.1 was used. Leaky ReLU activations with a leak rate of 0.1 were used for all models as was weight decay with a tuning parameter of 0.01. For the imposed prior a multi-variate standard normal distribution was used. For all optimizations, Adam was used with a learning rate automatically determined by the algorithm outlined by Smith (2017). The objective function, given in Eq. 1,

$$L\left(\theta_{e},\theta_{p};x_{i}\right)=-D_{KL}\left(p_{e}\left(z_{i}|x_{i};\theta_{e}\right)\;\|\;p_{p}\left(z_{i}|x_{i};\theta_{p}\right)\right)+E_{p_{e}\left(z|x_{i};\theta_{e}\right)}\left[\log p\left(x_{i}|z;\theta \right)\right]$$ (1)

minimizes the evidence lower bound by evaluating the weighted sum of the reconstruction loss and KL-divergence loss. However, due to the sampling step between the encoder output and the latent representation (Figure 5), this objective cannot be backpropagated through without employing the reparameterization trick. To do this, we modelled the latent distribution as the linear expression (Doersch, 2016),

$$\mu +\nu e^{\frac{1}{2}\log\left(\text{diag}\left(\Sigma \right)\right)}$$ (2)

where $\nu ∼e^{\frac{1}{2}\log\left(\text{diag}\left(\Sigma \right)\right)}$ acts as a dummy sample that corresponds to a sample that would otherwise have been drawn directly from the encoding distribution *p*_*e*_(**z**| ***μ***, **Σ**).

Detailed training information for all VAE models can be found in the logs at the companion repository under the github.com/brahste/novelty-detection.

#### 2.3.4 Adversarial autoencoder architectures

Adversarial autoencoders (AAEs) also have the advantage that—due to the nature of adversarial training—they can be interpreted probabilistically. As the latent space is constrained to mimic the distribution imposed upon it, with AAEs, alternative novelty ranking approaches are available beyond the conventional reconstruction-based approach. To explore these features of AAEs, and how they compare to other algorithms, two AAE architectures have been implemented in this work. Maintaining consistency with the previously mentioned CAE and VAE architectures, a Baseline AAE was built. The rationale behind this is driven by the desire to explore the extensibility of AAEs for novelty detection while sharing enough common ground between CAE and VAE counterparts to allow for meaningful comparison. By keeping the convolutional operations similar between one of the AAE architectures and one of the CAE and VAE architectures, differences in performance can be attributed to differences in the fundamental structure and training regime.

**FIGURE 6:**
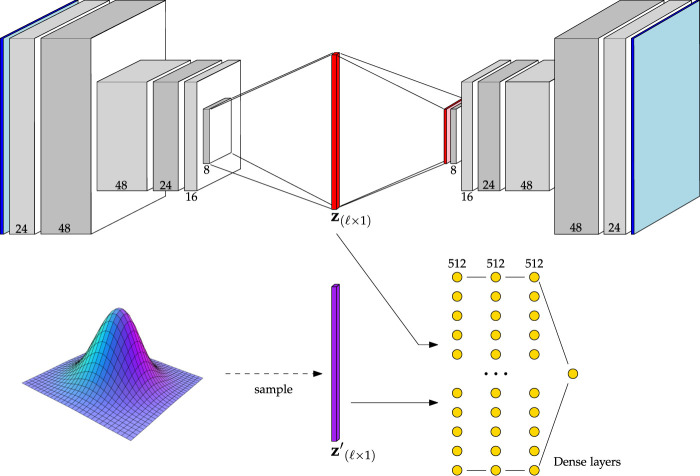
Architecture of the Baseline AAE. Channel numbers are shown at the bottom of each block. Reference Table 2 for a detailed description.

A graphical demonstration the Baseline AAE is shown in Figure 6. As can be seen from this figure, the convolutional structure of the Baseline AAE is the same as that outlined in Table 2. The only difference is found upon reaching the latent space. Here, its representation is flattened and passed either to the decoder or discriminator, depending on the optimization step.

The other AAE architecture, the Simple AAE, is a fully-connected alternative to the Baseline AAE, and was built to understand the implications of convolutional operations on AAE-style networks. The structure of the Simple AAE is replicated from the original paper on AAEs Makhzani et al. (2015).

Similar to the above autoencoder architectures, the Baseline AAE was trained with Leaky ReLU activations with a negative slope of 0.1, dropout with a drop rate of 0.1, and batch normalization with a momentum of 0.1. Weight decay with a tuning parameter of 0.01 was also used in both AAEs to reduce the range of obtainable weights, and thus regularize the network. As multiple optimizers have to be used to conduct adversarial training and the automatic learning rate determination algorithm is only realizable with single optimizer training, learning rates were set manually for all AAE implementations; values ranged between 0.01 and 0.0001. Early stopping was used with patience of five epochs. Batch sizes differed between datasets; a batch size of 256 was used when training on the Curiosity Mastcam data, and a batch size of 32 was used for the Lunar Analogue data.

The adversarial training regime uses three optimization steps. A variant of the Adam optimizer that decouples the weight decay from Adam’s natural gradient descent algorithm was used for all optimizations (Loshchilov and Hutter, 2019). Using an alternating optimization strategy, training commenced in two phases: the reconstruction phase and the regularization phase. First, in the reconstruction phase, a loss was computed by evaluating the mean squared error between the input and the reconstruction. Second, in the regularization phase, the parameters in the dropout and batch normalization layers of the encoder were frozen, effectively putting the model into inference mode. With the latent representation **z** computed by the encoder, a sample of the same size **z**′ was drawn from the imposed prior. Here, the prior was chosen to be a linearly independent (e.g. diagonal covariance matrix) multi-variate Gaussian with zero mean and unit standard deviation. Both samples were passed to the discriminator and the following loss was used as the optimization criterion for the discriminator,

$$-\frac{1}{C_{\ell }}\overset{C_{\ell }}{\underset{k=0}{\sum }}\left(\log,\left(D\left(z_{k}^{\prime }\right)+\epsilon \right),+,\text{log},\left(1-D\left(z_{k}\right)+\epsilon \right)\right).$$ (3)

In the above equation, *D*(⋅) denotes the function approximated by the discriminator, *C*_*ℓ*_ denotes the number of dimensions in both **z** and **z**′, and *ϵ* = 1 × 10^–8^ helps ensure numerical stability. By applying logarithmic laws and then recasting Eq. (3) as a maximization problem without the negative logarithms, one sees that an expectation is being taken over the core criterion,

$$D\left(z^{\prime }\right)\left(1-D\left(z\right)\right).$$ (4)

This expression can be maximized in one of two ways: by increasing the response of the discriminator to samples drawn from the prior (e.g. *D*(**z**′)), or by reducing the response of the discriminator to samples drawn from the encoder (e.g. *D*(**z**)). Hence, by optimizing over the objective in Eq. (3), the discriminator learns to recognize the source of each sample.

In the second step of the regularization phase, the discriminator is put into inference mode and the encoder is optimized alone via the criterion,

$$-\frac{1}{C_{\ell }}\overset{C_{\ell }}{\underset{k=0}{\sum }}\log\left(D\left(z_{k}\right)+\epsilon \right).$$ (5)

Again, considering the core quantity being optimized, *D*(**z**), this step trains the encoder to produce latent representations that are distributed similarly to the prior, in effect ambiguiating **z** and **z**′ in subsequent regularization phases.

This three-step optimization procedure, referred to herein as adversarial training, puts a responsibility on the encoder to simultaneously learn latent representations that are distributed according to the prior and that can be meaningfully reconstructed by the decoder.

The AAE implementations presented here mark the first use of such algorithms in the domain of planetary exploration to the best of the authors’ knowledge. Detailed training information for all AAE models can be found in the logs at the companion repository under the github.com/brahste/novelty-detection.

### 2.4 Evaluation metrics

When evaluating the performance of novelty detection algorithms it is important to use metrics that are applicable to such problems. One particularly powerful way novelty detection algorithms can enhance planetary surface analysis is through data prioritization. In an operational setting a certain false positive rate is inherent, especially in datasets with large amounts of uniformity. Still, there remains value in understanding the likelihood that a model’s prediction is correct, optimizing the model’s parameters to maximize confidence, and with such information, prioritize the data such that samples with higher confidence are parsed by human analysts first.

By definition, novelties are scarcely represented in the data, as such some evaluation metrics (such as accuracy) provide misleading assessments of performance. Others offer some benefit when measuring one variable, but become opaque when measuring another. Due to the complexities of real-world data, understanding the methods available to evaluate model performance, and subsequently selecting *specific models* for application-level software, is an important task. In the current study, simple and robust metrics derived from traditional model evaluation are employed (as discussed below). In addition, we propose a new metric—Precision at Capacity, denoted Precision(%-capacity)—that has proven invaluable when analyzing the performance of select models for application-level implementation (Stefanuk, 2021).

**FIGURE 7:**
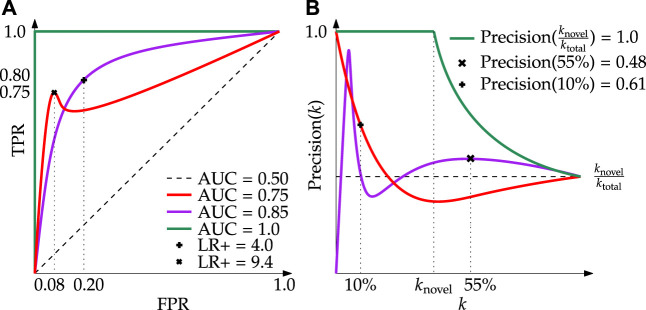
Demonstration of the core evaluation metrics used in this research. **(A)** Receiver Operating Characteristic (ROC) curves with associated AUC and LR+; here it can be seen that despite the purple line having a greater AUC, a well-selected model from the red line can achieve a LR+ >2× larger. **(B)** Precision(*k*) curves with representative Precision(% Capacity) values; here it can be seen that despite the purple line displaying better generic performance across the spectrum of *k* values, at 10% capacity the red line is more precise.

1) **Receiver Operating Characteristic (ROC)** curves are verbose graphical plots that display the performance of binary classifiers (Figure 7). An ROC curve plots the true positive rate (TPR), also known as recall, as a function of the false positive rate over a sequence of thresholds^2^. Novelty scores falling above the threshold are considered positive (novel) sample detections, those falling below are considered negative (typical) samples.
  i) **Positive Likelihood Ratio (LR+)** is a value that encodes the relative strength between the true positive rate (fraction of ground truth novelties correctly detected) and the false positive rate (fraction of typical samples erroneously detected). It is useful for application-level evaluation because it measures the likelihood that specific model parameters will result in the detection of more true positives than false positives, as weighted by the number of samples in each class.
  ii) **Area Under the Curve (AUC)** is a useful derived metric; in general, the greater the AUC, the better the performance. One drawback of ROC AUC is it generalizes performance across an array of thresholds, only implicitly providing insight into how well a fully implemented model would work in practice.
2) **Precision** **(k)** curves are graphical visualizations of precision (the fraction of positive detections that are correct) as a function of the number of samples, *k*, under test (Figure 7). More precisely, the plot displays the proportion of correctly identified novel samples given the *k* top-ranked samples. For example, with a model that always predicts a sample is novel,

$$\text{Precision}\left(k=2\right)=\left\{\begin{array}{cc} 1,\; & \text{if }x_{1}\wedge x_{2}\\ 0.5,\; & \text{if }x_{1}⊻x_{2}\\ 0,\; & \text{if }\neg \left(x_{1}\|x_{2}\right) \end{array}\right.$$ (6)

where *x*_1_ and *x*_2_ are the two most novel samples as predicted by the model.

i) **Precision(%-capacity)** is a metric developed by the authors that describes the precision of a select model at a distinct cut-off of the total capacity^3^. In this sense, the term capacity refers to the data transmission capacity: the percentage of data collected that can realizably be downlinked in a given period. For example, say 100 MB of data is collected in a day but due to transmission limitations, only 10 MB (10%) can be downlinked. This means that the data transmission capacity is 10% of the total data collected, thus Precision(10%) becomes the quantity of interest. In Figure 7 one can see the implications of this quantity for two example Precision(*k*) curves. Contrast this to the calculation of Precision(*k*), whereby a value is determined at increasing integers of *k* without regard for the size of the dataset at large. Precision (%-capacity) bridges another conceptual gap reported in literature (Xu et al., 2019; Kerner et al., 2020) whereby only samples up to the known number of novelties are used. To remain consistent with other works we also report this value as Precision(*k*_novel_); however, this metric relies on the assumption that the number of novelties can be known a priori. By their nature, novelties are uncommon, found at irregular intervals, and comprise a small, unknown fraction of the data collected. Precision(%-capacity) clarifies the attainable performance of a detector in bandwidth constrained environments where only a portion of accumulated data can be downlinked over a given duration.

## 3 Results and discussion

### 3.1 Curiosity mastcam

A state-of-the-art benchmark for novelty detection in a Martian context is a CAE that achieves an ROC AUC of 0.65 (Kerner et al., 2020). As stated in the introduction, one of the primary goals of this research is to investigate systems that are able to outperform this score. Results from this dataset can provide insights into how novelty detection is influenced by features specific to images of the Martian surface.

**FIGURE 8:**
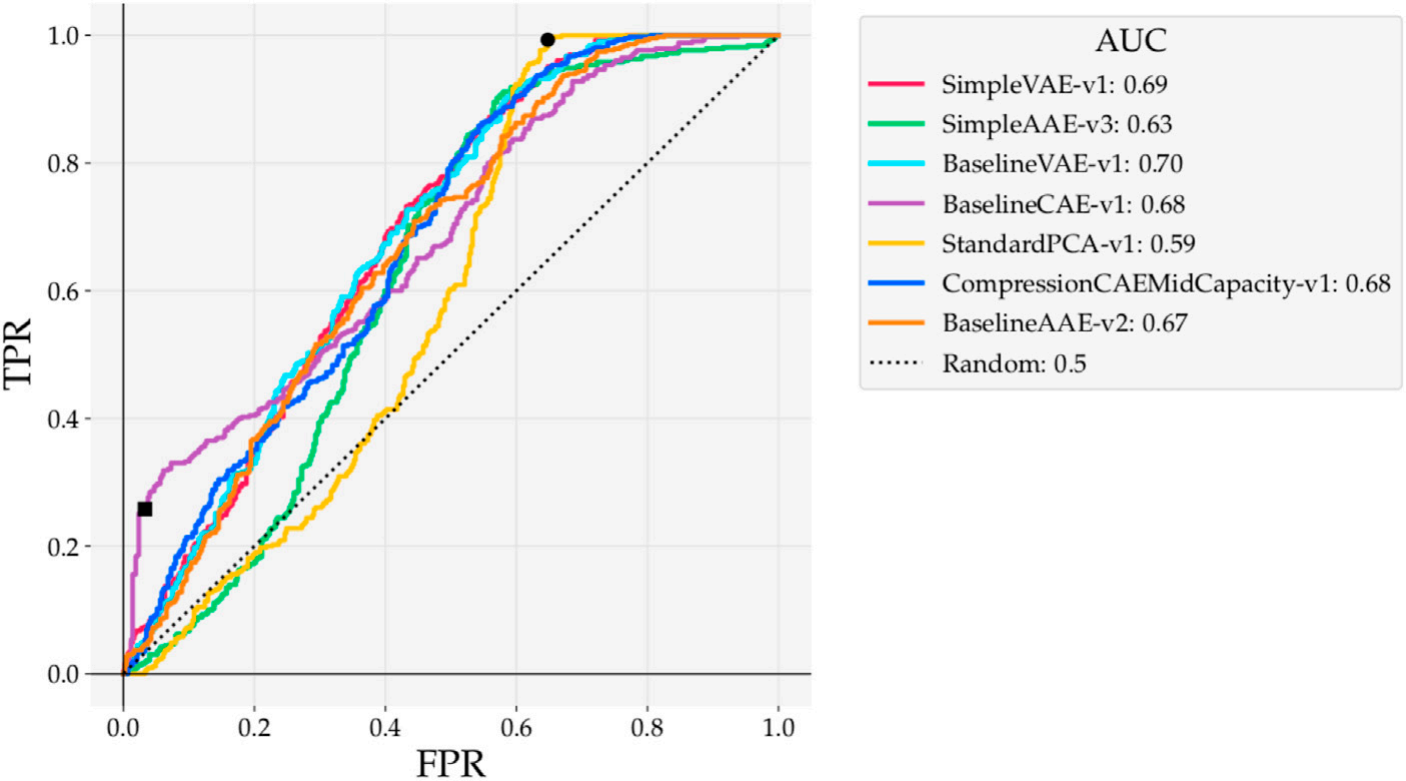
ROC curves for reconstruction-based detections with models trained on Curiosity Mastcam. At the point marked ■ the Baseline CAE obtains an LR+ = 7.85. At the point marked • the PCA model hits full recall earlier than any of the other models.

**FIGURE 9:**
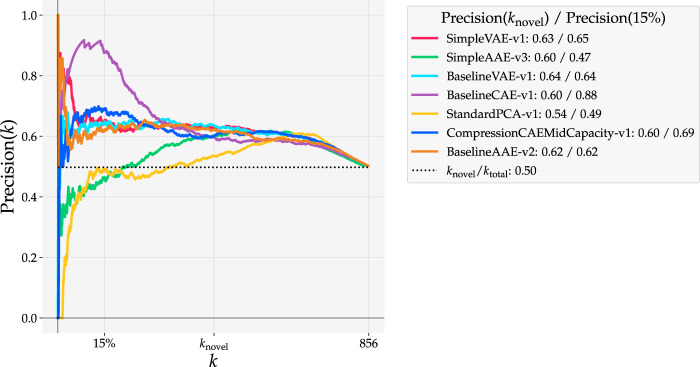
Precision(*k*) curves for reconstruction-based detections with models trained on Curiosity Mastcam. *k*_novel_ = 430 is the number of ground truth novel samples in the dataset and 15% of the data accounts for 128 samples.

**FIGURE 10:**
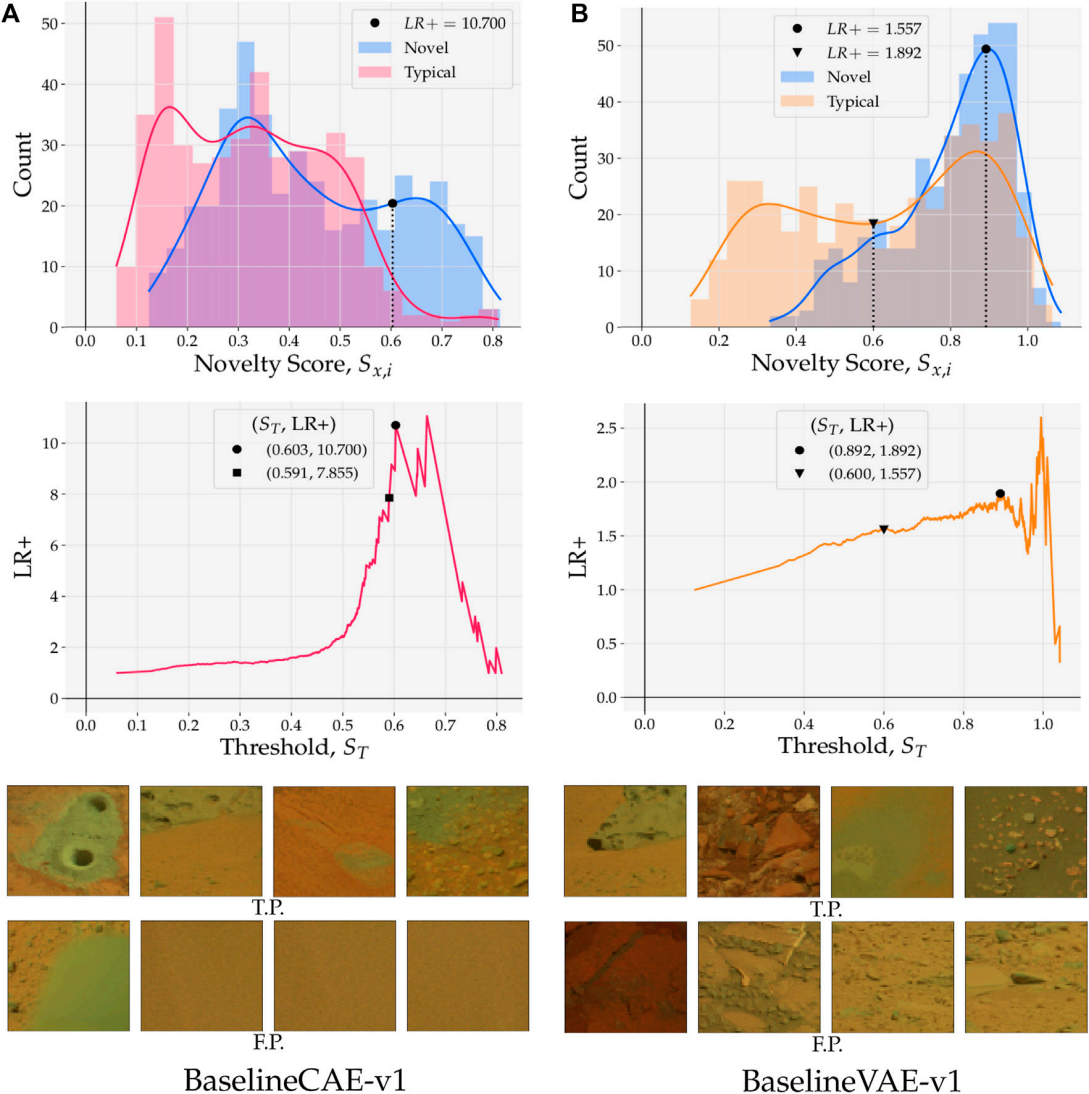
(*top*) Reconstruction score distributions for typical and novel samples; approximated probability densities were calculated with Gaussian kernel density estimation. Thresholds *S*_*T*_ placed on novelty scores *S* establish a boundary that separates positive (novel) and negative (typical) class predictions. (*middle*) Positive likelihood ratio (LR+) as a function of novelty boundary/threshold *S*_*T*_. LR+ provides information about the relative strength of the true positive rate against the false positive rate. (*bottom*) Examples of true positive (TP) and false positive (FP) samples predicted by models with *S*_*T*_ set at the point marked by •. **(A)** BaselineCAE-v1. **(B)** BaselineVAE-v1

Models trained on the Curiosity Mastcam dataset are evaluated here using reconstruction-based detections. Scores are assigned as the mean squared error (MSE) between the reconstruction and input. ROC curves for these models are shown in Figure 8 while Precision(*k*) curves are shown in Figure 9. To better understand the nature of the novelty scoring mechanism underpinning the performance of different models, in Figure 10 we also plot the distributions of novel and typical samples according to the novelty score assigned to them.

As measured by ROC AUC, the two VAEs performed the best (0.70, 0.69) while PCA performed the worst (0.59). ROC curves from this experiment are particularly interesting to observe because of the variety of shapes they take. For example, the PCA ROC curve performs nearly the same as random until False Positive Rate (FPR) ≈ 0.40, capturing almost all of its AUC afterwards. Despite its lower AUC, the peak of the PCA ROC curve hits full recall at FPR ≈ 0.65 (point marked by •), earlier than any of the other models. This means that, at the novelty boundary that results in FPR ≈ 0.65, the PCA detector correctly identifies all of the ground truth novelties with fewer false positives than any of the other models.

The bulge at the bottom left of Figure 8 resulting from Baseline CAE marks another interesting observation (point marked by ■). The Positive Likelihood Ratio (LR+) can be used to mark thresholds that return good, balanced detections. When selecting a detector to use in an operational scenario, this ratio is important because it identifies the performance of a *single* realizable detector. This metric can also be interpreted as the derivative of the ROC curve. With a LR+ = 7.85 at the point marked ■ in Figure 8, any predicted novel sample has a high confidence of being truly novel.

The Precision(*k*) curve (Figure 9) also shows a salient bulge when evaluating Baseline CAE at 15% data capacity. While the Precision(*k*_novel_) is highest for Simple VAE at 0.63, Precision(15%) is much higher with Baseline CAE than any of the other models at 0.88. More precisely, when detecting with Baseline CAE the highest 15% of novelty scores are associated with 88% of the ground truth novelties. In practice it is impossible to know *k*_novel_ beforehand. Since data downlink limitations are driven by means entirely unrelated to the number of novelties, Figure 9 proves the value of assessing the precision at a specified data capacity, a value that is driven by operational factors such as bandwidth constraints and line of sight opportunities.

Class-wise results for the Baseline models trained on Curiosity Mastcam are provided in Table 3. Baseline VAE not only obtains the best ROC AUC overall (Figure 8), but also exhibits more consistent AUCs across novelty classes than either the CAE or AAE. These results contrast the technically superior RX detector from (Kerner et al., 2020) that achieved an ROC AUC of 0.72, but showed high volatility when detecting on a class-wise basis (AUC 0.22 detecting veins versus AUC 0.97 when detection meteorites), indicating that RX detectors are susceptible to specific types of spectral and morphological features and that Baseline VAE is superior in terms generic novelty detection. Moreover, RX detectors were not selected as state-of-the-art for comparison in this work because they do not generalize to 3-channel colour images, which are the only ones currently available for lunar applications. Once multi- or hyper-spectral lunar surface images become available, RX detectors should be given a more detailed treatment to fully understand their capabilities in the lunar environment.

**TABLE 3:** Class-wise ROC AUCs for Baseline models trained on curiosity Mastcam.

Novelty class	Baseline CAE	Baseline VAE	Baseline AAE
All	0.684	**0.697**	0.671
Bedrock	0.443	**0.581**	0.552
Broken rock	0.521	0.719	**0.725**
Drill hole	**0.751**	0.640	0.608
DRT spot	**0.857**	0.743	0.725
Dump pile	**0.752**	0.666	0.639
Float	0.571	**0.660**	0.626
Meteorite	0.549	**0.619**	0.594
Vein	0.416	**0.692**	0.686

The bold values refer to the best ROC AUC for each of the novelty classes.

 in Figure 10 (top), novelty scores from test samples are binned and coloured by their ground truth label. A probability density estimate is then computed using Gaussian kernel density estimation (KDE) with kernel $N\left(S;\sigma^{2}\right)$ where *σ* = 0.55, as determined by Scott’s method (Scott, 1992). In Figure 10A (top) it can be seen that novel samples dominate the distribution above the right-most KDE intersection at *S* = 0.548. This linear separability gives context as to why the BASELINE CAE-V1 shows high precision at low data capacity in Figure 9 ^4^. Since models exhibiting high precision are particularly valuable for data prioritization (e.g. in a bandwidth constrained environment), the positive likelihood ratio (LR+) plays a special role when selecting a specific model because it provides information about the relationship between the true positive rate and the false positive rate. In Figure 10A (middle), LR+ is plotted as a function of novelty thresholds. It is observed that for the Baseline CAE, LR+ hits a local maximum at the point marked by • with (*S*_*T*_, LR+) = (0.603, 10.700). A model chosen with this threshold would yield a TPR 10.7 × higher than its FPR. While there does exist a global maxima at *S*_*T*_ = 0.664, such a threshold would omit 42 true positives to increase LR+ to 10.900; if the ultimate goal is to reduce false positives at all costs, such a trade-off may be desirable, but is left for the reader to decide Such results demonstrate that performant, domain-specific models with low FPRs can be built and selected using existing statistical metrics and a small amount of qualitative assessment.

Figure 10A (*bottom*) shows randomly selected examples of true positives and false positives that were reported by the model with *S*_*T*_ = 0.603. Interestingly, all of the false positives are highly homogeneous with little or no morphological structure.

Switching to Figure 10B where results pertaining to the Baseline VAE are shown, it is observed that both the novel and typical distributions exhibit at least one mode in the region above *S*_*T*_ = 0.600. Overlap such as this is a sign that the predictions given by the model lack separability in high scoring regions. Nonetheless, the distribution of typical samples shows bi-modal behaviour (as opposed to the novel samples which show uni-modal behaviour) with a large fraction of its scores being concentrated in the lower region where *S* < 0.600 (point marked ▾). Again using the LR+ curve (Figure 10B (*middle*)), we see a local maximum at *S*_*T*_ = 0.892 (marked by •) and global maximum at *S*_*T*_ = 0.992. Although the latter threshold obtains a better LR+, in the vicinity of *S*_*T*_ = 0.992 the curve is noisy due to the small number of samples with *S* > *S*_*T*_. In an operational scenario, a model that implements *S*_*T*_ = 0.870 will hence provide a more robust solution at the expense of LR+. Such a model is recall-dominant and is best applied when seeking to maximize the number of ground truth novelties detected. In these scenarios, on-board computation is less valuable than the counterpart shown in Figure 10A since its data prioritization potential is low. However, recall-dominated models that lack precision are still valuable on-ground as a first-pass filtering mechanism that helps resolve delayed tactical cycles (Gaines et al., 2016).

The observations outlined herein show that when inferring novelty on a linear scale of scores, measures such as ROC AUC and Precision(*k*_novel_) are insufficient to draw informed conclusions about relative performance. For example, while both Baseline CAE and Baseline VAE obtain a similar ROC AUC, these values are arrived at in two distinct ways. One way is to produce higher proportions of novel to typical samples in the region where *S* is high (Figure 10A), the other is to produce lower proportions of novel to typical samples in the region where *S* is low (Figure 10B). Despite the ROC AUC and Precision(*k*_novel_) scores identifying similar performance, a deeper analysis of a models’ underlying properties, including probability density approximations and positive likelihood distributions, makes the model selection process more transparent and less qualitative.

**FIGURE 11:**
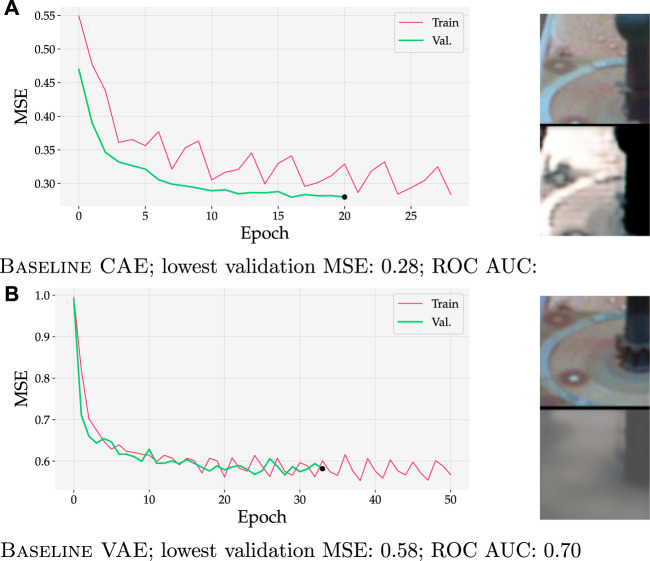
Training and validation curves (left) for two models trained on Curiosity Mastcam data. Input and reconstruction samples from the final validation epoch are shown for qualitative comparison. Although MSEs were almost 2× higher for Baseline VAE, it still achieved a slightly better ROC AUC than Baseline CAE. **(A)** Baseline CAE; lowest validation MSE = 0.28 achieves an ROC AUC = 0.68. **(B)** Baseline VAE; lowest validation MSE = 0.58 achieves an ROC AUC = 0.70.

Figure 11 demonstrates that VAEs can cope with higher MSE scores while retaining state-of-the-art performance. In this figure, the MSE of Baseline VAE trained on Curiosity Mastcam at the final validation epoch was calculated to be 0.58 (Figure 11B, marked with •). Contrarily, a lowest validation loss of 0.28 was obtained with Baseline CAE (Figure 11A). The obvious qualitative nature of this difference can be observed in the inputs and reconstructions in the rightmost images. When detecting with Baseline VAE, the reconstructed image loses fine features such as the ring and circular spots surrounding the calibration target (dark pole with knob on top). Though a greater level of detail is kept with Baseline CAE (as also evidenced by its lower MSE), the general performance as measured by ROC AUC is marginally higher for Baseline VAE.

### 3.2 Lunar analogue

**FIGURE 12:**
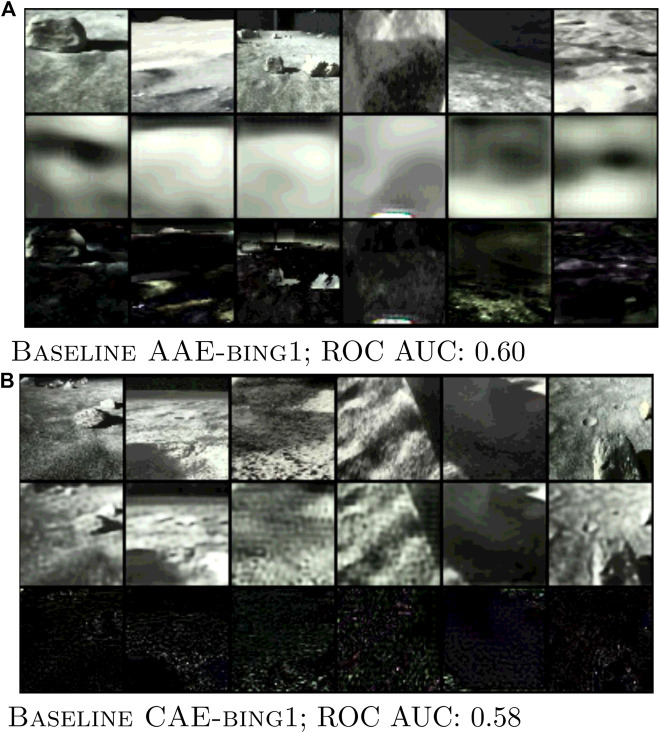
Images of inputs (top), reconstructions (middle), and error maps (bottom) obtained during training for two novelty detection models. Notice the objectively poor reconstructions in **(A)** BaselineAAE-bing1 achieving an ROC AUC=0.60. **(B)** BaselineCAE-bing1 achieving an ROC AUC=0.58.

The findings in the previous section on Curiosity Mastcam data suggest that the performance of a novelty detector is not directly driven by the ability to reconstruct typical images *well*. Instead, it is driven by the relative ability to reconstruct typical images *better* than novel ones, even at the expense of objectively poor reconstructions. We can observe further examples of this phenomenon in Lunar Analogue data shown in Figure 12, where inputs, reconstructions, and normalized error maps are displayed. To be precise, each error map pixel is taken as the squared difference between the input **x** and reconstruction $\hat{x}$ such that,

$${\text{error}}_{i,j}={\left(x_{i,j}-{\hat{x}}_{i,j}\right)}^{2}$$ (7)

Inputs were obtained by randomly sampling six images from the first training batch of the lowest validation epoch (with patience of five epochs). As is seen in Figure 12B the mean intensity in the error maps of Baseline CAE are lower than for Baseline AAE (Figure 12A). One may also notice that the reconstructions shown in Figure 12A have a lower frequency than those in Figure 12B, omitting much of the details in the reconstruction. Remarkably, the ROC AUC of Baseline AAE is marginally higher.

## 4 Conclusion

Well-tuned autoencoders, be they CAEs, VAEs, or AAEs, provide promising performance. On Curiosity Mastcam data the ROC AUCs of all Baseline architectures showed performance within 0.685 ± 0.015, with the best AUC being found with Baseline VAE (0.70). The previous state-of-the-art presented by Kerner et al. (2020) achieved an ROC AUC of 0.65, and only a max ROC AUC of 0.61 when using MSE metrics. Therefore, this Baseline VAE model presented in this work outperforms the previous state-of-the-art by 0.70/0.65 = 1.077, or >7% , and outperforms the MSE-only reference model by 0.70/0.61 = 1.148, or >14% . Moreover, when analyzed according to Precision(15%) the Baseline CAE achieved a precision of 0.88. This important result proves that when trained on a representative dataset, the correct algorithm can boost the proportion of novel to typical samples considerably while retaining good recall (see Figures 8, 9). Lastly, these results were obtained within the scope of a single objective and novelty scoring function: mean squared error. Our supplementary findings published in (Stefanuk et al., 2020) showed that the best results were obtained by using a hybrid SSIM/MSE objective function and Outlier Count as the novelty scoring function. By extending the algorithms presented in this paper to use alternative optimizations and operations, performance could be improved even further. Such investigations were omitted here so that focus could be placed on widening the scope of available autoencoder algorithms, all of which can be routinely extended to use different operations such as SSIM or Outlier Count.

Another important aspect of this study is the use of VAEs and AAEs. Until recently, VAEs and AAEs had been primarily considered for novelty detection on datasets similar to Novelty MNIST (An and Cho, 2015; Leveau et al., 2017; Beggel et al., 2019). Our research took these algorithms and applied them to complex real-world data, demonstrating their capabilities in both academic and commercial realms. In certain scenarios, it was shown that the implemented VAEs and AAEs outperform more conventional techniques, such as PCA. Furthermore, they enable structured latent representations, which could further enable the meaningful utilization of the latent space for direct novelty detection. Thus with VAEs and AAEs rich alternatives to reconstruction-based detections become available.

### 4.1 Closure on precision and recall

When the connection between precision and recall is interpreted in the context of planetary exploration, important insights arise regarding what to look for in high performance novelty detectors. In our experiments, ROC curves were used to understand the recall of a model while Precision(*k*) curves displayed information about precision. Whether a detector is viewed through the lens of precision (e.g. Figure 9) or the lens of recall (e.g. Figure 8) affects the interpretation of its performance.

Fundamentally, when evaluating the recall (or true positive rate) of a detector, what we consider is: “Of all the ground truth novelties present in the dataset, how many are correctly identified as novel.” Alternatively, when evaluating the precision (or positive predictive value) of a detector, what we consider is: “Of all the predicted novelties, how many are ground truth novel?”

Detectors that achieve high recall are best suited for scenarios where it is most important to catch every novelty. For these detectors, the amount of samples incorrectly identified as novel is a secondary concern; the detector’s job is in this case is to prune the obviously typical samples to reduce overall workload. Such scenarios dominate current approaches to planetary novelty detection on-ground because it is easy to conduct high-throughput image analysis on already downlinked data, when bandwidth constraints are no longer a concern. However, recall must not be used as a sole metric, as simply labeling all images novel achieves perfect recall. This is why ROC curves were used as a primary evaluation metric in this work, as they visualize the trade off between recall (TPR) and false positive rate. Most of our experiments showcased examples of high recall detectors. All autoencoder models trained on Curiosity Mastcam (Figure 8) obtained recalls of 0.60 at FPR = 0.38 ± 0.03. When trained on Lunar Analogue region proposals (Figure 12) the Baseline CAE and Baseline AAE obtained recalls of 0.6 at FPR = 0.35 ± 0.02. All of these models are well-suited for on-ground processing to boost the ratio of novel to typical samples.

Detectors with good precision are best suited for scenarios where it is most important to maximize the proportion of correctly identified novelties from a subset of images. For these detectors, missing large numbers of novelties altogether is acceptable. Bandwidth constrained environments exemplify a particularly valuable application of high precision detectors since the most novel data can be identified and prioritized for downlink. Unfortunately, bandwidth constrained environments are often also constrained in terms of computational resources. Hence on-board pre-filtering may be avoided for reasons related to power availability or hardware limitations. As a metric, precision also has the disadvantage that it only considers novel predictions in its evaluation, so it fails to incorporate information about how many ground truth novelties were overlooked entirely.

A visual example of precision and recall in planetary novelty detection is illuminated in Figure 10. Imagine a threshold is placed at *S* = 0.6 for both Baseline CAE a) and Baseline VAE (b). For Baseline CAE this threshold would produce a high precision detector because most of the typical and novel samples would be omitted, but those that were retained would be almost entirely novel. On the other hand, this threshold placed on Baseline VAE would produce a high recall detector since most of the novelties would be recognized, but many typical samples would be mistaken for novelties as well.

Unlike recall, which is governed by the number of ground truth novelties and ranges between zero and one accordingly. As such, when using precision to evaluate performance, the goal post should be set to *k*_novel_/*k*_total_ as opposed to some static value such as AUC = 0.5 for ROC curves. If precision is higher than *k*_novel_/*k*_total_ and computational concerns are not an issue, then using a detector for on-board pre-filtering is a good idea.

Since each of the two metrics have their place in specific scenarios, neither can objectively be called better. Under certain conditions, however, one may be more appropriate as the basis of evaluation, and both should always be used to counter-balance conclusions made from the other.

### 4.2 Dimensionality reduction and quality of reconstruction

Many factors are at play when switching between algorithms and training regimes. One of the primary reasons why the reconstructions resulting from the AAE are objectively poor compared to the CAE is that the latent representation is lower dimensional. The latent representation of Baseline AAE-bing1 occupies $R^{32}$ , the latent size of Baseline CAE occupies $R^{768}$ , 24-times higher in dimension. In other words, the latent representation undergoes a larger dimensional reduction when encoded by the AAE. It appears that there is a “Goldilocks Zone” for dimensional reduction that results in the best performance. The key insight here is that this zone does not arise because certain levels of compression provide good reconstructions, but because they lose *just enough* information that the model of normality learns only the most dominant typical features. At the most appropriate compression, upon reconstruction, features that are reminiscent of novelties (whether they be typical or not) are lost. While guiding design decisions based on this interpretation may omit some typical content in the reconstruction of typical images, such a concern is secondary under the presumption that novel images are relatively devoid of typical content. Thus, when they are mapped onto the learned typical modes of the model, most of the novel content will fail to be reconstructed, resulting in higher MSE scores.

As shown in Figures 11, 12, our research supports this interpretation because it demonstrates that VAEs and AAEs can cope with higher MSE scores while retaining state-of-the-art performance. Though a greater level of detail is kept with Baseline CAE (as evidenced by its lower MSE), the general performance as measured by ROC AUC remains marginally higher for Baseline VAE or Baseline AAE respectively.

For reasons mentioned above, these insights are most applicable to complex, real-world images that have fine, subtle details. Future novelty detection researchers applying their expertise to such data are advised to consider the interpretation presented herein—the best performing models are ones that prioritize the most dominant typical features. Models that return high quality reconstructions may suffer from overfitting, and are often capable of reconstructing novel features on-par with typical ones encountered during training.

### 4.3 Future work

Avenues for future research are plentiful, some of which have been mentioned throughout the text.

Perhaps the single largest factor limiting the development of general purpose novelty detection systems for planetary exploration is the lack of available data. While many sources and databases exist with images of the lunar and Martian surface (NASA, 2017; CNSA, 2021), very few research endeavours have gone through the process of preparing large, high-quality datasets with labels specifically designed to address novelty detection tasks. Currently, projects such as LabelMars are underway which will help alleviate this developmental bottleneck for martian-based systems. One important project that should be undertaken is to conduct similar measures as LabelMars but for lunar surface images, for example, LabelMoon. Expert analysis of existing lunar surface images is a valuable first line of attack.

In this research, all of the models used for novelty detection are optimized using a mean squared error (MSE) objective. For the reconstruction-based techniques, the MSE was also used to directly score the novelness of each input sample. In work published in the early stages of this research (Stefanuk et al., 2020) a wider array of operations were used both for the optimization objective (e.g., loss function) and novelty scoring function. Similar research was conducted along this avenue in the work by Kerner et al. (2020). In both these studies, the performance of MSE as an optimization objective between the reconstruction and input was compared and contrasted to the structural similarity index (SSIM), as well as a linear combination of MSE and SSIM. Though these measures mark the most transparent operations to optimize, there are potentially other techniques that could be used too. For example, expanding upon the linear combination approach, polynomial functions containing both SSIM and MSE could be valuable loss functions with little overhead required to adapt current training regimes. Our preliminary exploration of SSIM and hybridized loss functions have demonstrated that carefully selecting the optimization criterion can improve detection performance for reconstruction-based methods. In addition, Kerner et al. (2020) introduced the Outlier Count method, whereby the squared error of each pixel in the reconstruction and input image is computed.

When applying novelty detection to a specific domain, such as the Moon or Mars, (hyper) parameters must be adapted to suit the domain. Differences in illumination, distribution of features, colours, and general geologic content all affect the ability of a reconstruction algorithm to detect novel behaviour within an image, as do the size and spectral dimension (i.e. number of channels) or the image. Training parameters such as the learning rate, weight decay, network capacity, and model architecture are all impacted by the domain of interest, and will not necessarily show similar performance when applied to different environments. Considerations such as these are crucial when building a novelty detection system deployable to targeted environments. One promising direction of research lies in tuning a system to a specific environment to its fullest extent, such research would be highly valuable as commercial software products.

All of the detections carried out in this research use thresholds to establish novelty boundaries. In the current study, this approach was selected because, independent of the model used, more sophisticated binary classifiers can always be cascaded with them. This research outlines the potential for advanced autoencoder methods, such as VAEs and AAEs, to provide reconstruction outputs that in some cases perform better than previous state-of-the-art CAE methods. A CNN can be cascaded with any of these architectures to extract subtle features in the error map between input and reconstruction. As a logical extension to this, since VAEs and AAEs enable structured latent representations, it is possible to cascade a CNN with the latent representation to conduct intermediary detections to either supplement or replace reconstruction detections. End-to-end trainable networks are also of interest for image-based novelty detection. These networks would not require any cascaded networks, but would instead build the CNN classifier directly into the overall architecture, effectively creating a network that outputs novelty detections directly. An illuminating discussion on end-to-end trainable networks and their place in novelty/anomaly detection is given by Pang et al. (2020).

Another interesting way to further extend the research in this work is to develop better techniques for novelty detection using the latent representations made available with VAEs and AAEs. Since the latent space is effectively an embedding of a high-dimensional image into a low dimensional representation, any detection techniques that are prohibited because of the curse of dimensionality become available. Probabilistic and information theoretic detection methods may provide better performance than the distance measures used in our experiments. A good starting point would be to consult the comprehensive review given by Pimentel et al. (2014). Leveraging the latent space for image-based novelty detection has been considered with VAEs in previous works (An and Cho, 2015). One study has shown that ROC AUCs can be improved for both latent space and reconstruction-based detections (Sintini and Kunze, 2020).

## Data Availability

The datasets presented in this study can be found in online repositories. For details about accessing the datasets go to https://github.com/brahste/novelty-detection.

## Footnotes

1 In particular, NASA’s Martian rovers Spirit, Opportunity (Crisp et al., 2003), Curiosity (Bell et al., 2017), and Perseverance (Bell et al., 2021), as well as the CNSA’s lunar rovers Yutu-1 (Jia et al., 2016) and Yutu-2 (Jia et al., 2018).

2 In other words, a 1-dimensional novelty boundary.

3 Precision(%-capacity) is usually denoted with a percentage (e.g., Precision(10%)) or sometimes with a fraction/decimal (e.g., Precision(0.1).

4 For reference, the threshold marked by ■ that was heuristically chosen in Figure 8 is also plotted in Figure 10A (*middle*).
